# Prophylactic, single-drug cardioprotection in a comparative, experimental study of doxorubicin-induced cardiomyopathy

**DOI:** 10.1186/s12967-020-02564-w

**Published:** 2020-12-09

**Authors:** Mária Lódi, Viktor Bánhegyi, Beáta Bódi, Alexandra Gyöngyösi, Árpád Kovács, Anita Árokszállási, Nazha Hamdani, Miklós Fagyas, István Édes, Zoltán Csanádi, István Czuriga, Zoltán Kisvárday, István Lekli, Péter Bai, Attila Tóth, Zoltán Papp, Dániel Czuriga

**Affiliations:** 1 Division of Clinical Physiology, Department of Cardiology, Faculty of Medicine, University of Debrecen, Debrecen, Hungary; 2 Kálmán Laki Doctoral School, University of Debrecen, Debrecen, Hungary; 3 Department of Pharmacology, Faculty of Pharmacy, University of Debrecen, Debrecen, Hungary; 4 Department of Oncology, Faculty of Medicine, University of Debrecen, Debrecen, Hungary; 5 Department of Molecular and Experimental Cardiology, Ruhr University Bochum, Bochum, Germany; 6 Department of Cardiology, St. Josef-Hospital, Ruhr University Bochum, Bochum, Germany; 7 Division of Cardiology, Department of Cardiology, Faculty of Medicine, University of Debrecen, Debrecen, Hungary; 8 Department of Anatomy, Histology and Embryology, Faculty of Medicine, University of Debrecen, Debrecen, Hungary; 9 MTA-DE Lendület Laboratory of Cellular Metabolism, Debrecen, Hungary

**Keywords:** Doxorubicin, Anthracycline, Cardiotoxicity, Animal model, Heart failure

## Abstract

**Background:**

Cardiomyopathy is a common side effect of doxorubicin (DOX) chemotherapy. Despite intensive research efforts in the field, there is still no evidence available for routine cardioprotective prophylaxis to prevent cardiotoxicity in the majority of oncological patients at low risk of cardiovascular disease. We have recently demonstrated the advantages of a prophylactic, combined heart failure therapy in an experimental model of DOX-induced cardiomyopathy. In the current work, we focus on individually applied prophylactic medications studied in the same translational environment to clarify their distinct roles in the prevention of DOX cardiotoxicity.

**Methods:**

Twelve-week-old male Wistar rats were divided into 5 subgroups. Prophylactic β-blocker (BB, bisoprolol), angiotensin-converting enzyme inhibitor (ACEI, perindopril) or aldosterone antagonist (AA, eplerenone) treatments were applied 1 week before DOX administration, then 6 cycles of intravenous DOX chemotherapy were administered. Rats receiving only intravenous DOX or saline served as positive and negative controls. Blood pressure, heart rate, body weight, and echocardiographic parameters were monitored in vivo. Two months after the last DOX administration, the animals were sacrificed, and their heart and serum samples were frozen in liquid nitrogen for histological, mechanical, and biochemical measurements.

**Results:**

All prophylactic treatments increased the survival of DOX-receiving animals. The lowest mortality rates were seen in the BB and ACEI groups. The left ventricular ejection fraction was only preserved in the BB group. The DOX-induced increase in the isovolumetric relaxation time could not be prevented by any prophylactic treatment. A decreased number of apoptotic nuclei and a preserved myocardial ultrastructure were found in all groups receiving prophylactic cardioprotection, while the DOX-induced fibrotic remodelling and the increase in caspase-3 levels could only be substantially prevented by the BB and ACEI treatments.

**Conclusion:**

Primary prophylaxis with cardioprotective agents like BB or ACEI has a key role in the prevention of DOX-induced cardiotoxicity in healthy rats. Future human studies are necessary to implement this finding in the clinical management of oncological patients free of cardiovascular risk factors.

## Introduction

Cancer is the second leading cause of death globally with alarmingly increasing incidence [1]. Despite the dramatic improvement of modern oncotherapy (immunotherapy, targeted therapy), conventional chemotherapeutic agents are still considered indispensable components of curative and palliative antineoplastic regimens. Anthracyclines (AC), including doxorubicin (DOX), are essential parts of chemotherapeutic combinations in haematological and solid malignancies (breast cancer, sarcomas, gynaecological cancers, etc.). However, various side effects, such as acute or chronic cardiotoxicity may limit the use of DOX in a dose-dependent manner [2, 3]. The administration of 500–550 mg/m^2^ intravenous DOX may cause myocardial dysfunction in approximately 4–26% of patients, while a dose increase to 551–600 mg/m^2^ may lead to a prevalence of 18–26%. When the dose of DOX exceeds 600 mg/m^2^, the risk of cardiotoxicity can be as high as 36–48% [4–6].

Complex preventive strategies are necessary to reduce the cardiotoxic side effects of DOX, however, recent consensus statements and position papers have justified the primary application of cardioprotective pharmacological therapy only in the case of high-risk patients [2, 3, 7, 8]. In the past few years, several human and animal studies have been conducted in the hope of developing an effective strategy against DOX cardiotoxicity. These efforts include the relative risk evaluation of oncological patients: DOX-induced cardiomyopathy can occur more frequently in patients undergone previous radiation therapy or earlier AC treatment, as well as in elderly patients (> 65 years), and in paediatric populations (< 18 years). Other factors, such as genetic background, female gender, previous cardiac dysfunction, or hypertension may also lead to an increased risk of DOX cardiomyopathy [3]. Limiting the maximum cumulative dose of DOX to 450–550 mg/m^2^ may eliminate acute and early-onset chronic myocardial deterioration, but it has a poor impact on late-onset chronic cardiotoxicity [6, 9, 10]. Using AC analogues, such as epirubicin, also failed to reveal any positive effects due to a similar degree of cardiotoxicity at higher doses (> 900 mg/m^2^) [11, 12]. Promisingly, liposomal or pegylated DOX seem to be more protective against cardiomyopathy, while showing similar therapeutic efficacy as normal DOX [13–15]. However, these formulations are restricted to monotherapy or a limited number of combined chemotherapeutic protocols. The use of cardioprotective medications, such as probucol, N-acetyl cysteine, or dexrazoxane, have been previously tested in experimental and clinical studies, but only dexrazoxane was found to be a promising agent to attenuate cardiotoxicity [16–27]. Although many reviews have suggested the prophylactic use of the iron-chelator dexrazoxane for cardioprotection, some human studies have revealed a higher frequency of the haematological side effects of this drug (leukopenia, neutropenia, thrombocytopenia, etc.) in patients receiving AC therapy supplemented with dexrazoxane [28–30]. In the past, several heart failure (HF) medications (β-blockers, inhibitors of the renin–angiotensin–aldosterone system, lipid-lowering agents) were also examined in both animal models and human trials with varying success rates [31–39]. Most of these medications demonstrated an antioxidant effect to some extent. In addition, they did not interfere with the antitumor activity of DOX.

Despite the above described research efforts, still no firm evidence for a routine, widespread, primary preventive approach for DOX-induced cardiotoxicity has been incorporated into clinical practice guidelines [2, 3, 7, 8]. In this regard, one missing piece of information could originate from the inadequate experimental setting previously used in many rodent models, where several clinical aspects of the applied oncotherapy and the cardioprotective treatments were disregarded (e.g. a single high dose instead of consecutive cycles of DOX, intraperitoneal instead of intravenous DOX administration, cardioprotective drug in the drinking water supply instead of oral gavage, a short follow-up period, etc.) [40, 41]. Thus, it has been recently recommended that future preclinical cardioprotective studies for AC cardiotoxicity should more appropriately mimic the human pathology by the use of cyclic, intravenous AC, a longer study follow-up period, and the application of the cardioprotectant regimen prior to the AC chemotherapy. It was also recommended to use both female and male rodent animals to study the gender differences in the prevention of DOX cardiomyopathy [41]. Although the female gender is a risk factor in the development of DOX cardiomyopathy in humans, male rodents are more sensitive to AC exposure compared to females [10, 41–48].

In line with the above recommendations, we have recently demonstrated the effectiveness of a prophylactic, triple-combined HF therapy vs. the same treatment applied only at a later stage in a rat model of DOX cardiomyopathy, where our experimental design closely mimicked current human oncotherapeutic and drug interventional protocols [40]. In our present study, we focus on the effects of prophylactic, individually applied drug treatments using the same HF medications as in our previous model [β-blocker (bisoprolol; BB), angiotensin-converting enzyme inhibitor (perindopril; ACEI), and aldosterone antagonist (eplerenone; AA)] in order to clarify their distinct roles in the prevention of DOX cardiotoxicity.

## Methods

### Animal experiments and study design

**Fig. 1:**
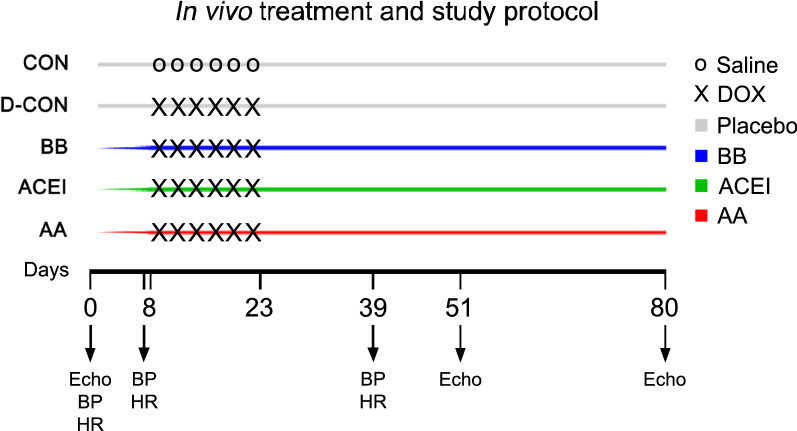
In vivo study protocol. Following baseline BP, HR measurements, and echocardiography, animals in the BB, ACEI, and AA groups received daily bisoprolol, perindopril, and eplerenone, respectively. Animals in the CON and D-CON groups received an oral drug-free vehicle (“placebo”) daily. DOX administration was performed on experimental days 8, 11, 14, 17, 20 and 23 in the D-CON, BB, ACEI, and AA groups. Animals in the CON group received intravenous saline on the same days. Repeated BP and HR measurements were performed on days 7 and 39, while follow-up echocardiography was carried out on days 51 and 80. AA = aldosterone antagonist (eplerenone), ACEI = angiotensin-converting enzyme inhibitor (perindopril), BB = β-blocker (bisoprolol), DOX = doxorubicin, Echo = echocardiography

Our in vivo protocol is presented in Fig. 1. In our experiments, we closely mimicked human chemotherapy protocols by administering DOX in 6 consecutive intravenous cycles at a concentration of 1.5 mg/kg into the tail vein of the animals. Negative control animals (CON) received intravenous saline instead of DOX on the same days. Prophylactic medications were applied by oral gavage on a daily basis, always at the same time during the day. DOX administration, blood pressure (BP), and heart rate (HR) measurements, as well as echocardiography were performed as described earlier [40]. We used 12-week-old male Wistar rats (*n* = 8–12 animals per group) for the study. BP and HR were monitored on experimental days 0, 7 and 39. Echocardiography was performed in deep anaesthesia of ketamine:xylazine combination (100 mg/kg ketamine, 10 mg/kg xylazine) on days 0, 51 and 80. The animals were divided into 5 subgroups. Prophylactic treatments of bisoprolol (2.5 mg/kg; BB), perindopril (2 mg/kg; ACEI), and eplerenone (6.25 mg/kg; AA) were started a week before the DOX treatment, while the animals serving as positive (D-CON) and negative controls (CON) received a drug-free vehicle orally. Following echocardiography on day 80, the animals were anaesthetised using intraperitoneal thiopental (100 mg/kg), their hearts were excised, frozen in liquid nitrogen, and stored at − 70 °C. Lungs of the animals were also removed, and weighed before and after a drying process at 60 °C for 24 h. Native blood samples were centrifugated, then sera samples were frozen and stored at − 70 °C.

### Echocardiography

Echocardiography measurements were carried out using a General Electric Vivid E9 ultrasound system equipped with a linear 14.1 MHz i13L probe (General Electric, Fairfield, CT, USA). For M mode-based systolic parameters the parasternal long axis view, for diastolic and Doppler-based systolic parameters the 4-chamber view was examined. To investigate strain parameters, a short cine loop was acquired from the 4-chamber view. Due to strict criteria of image quality, only two segments of the septum (basal and mid) were used for the assessment of strain parameters. Echocardiography images were acquired along with continuous electrocardiogram recording (limb leads).

### Histology

The detailed protocol of the histological assessment was described earlier [40]. Briefly, 15 µm sections were cut from the left ventricles of the rats using Cryotome™ Cryostat (Thermo Fisher Scientific, Waltham, MA, USA) and were stained with Mayer’s hemalum (VWR International, Radnor, PA, USA) and picrosirius red. The sections were then dehydrated in grading series of ethanol, mounted with DPX (Sigma Aldrich, MO, USA) and investigated under an Olympus BX-50 microscope. The fibrotic area and the capillary density were analysed on the basis of representative images using the ImageJ (National Institutes of Health, Bethesda, Maryland, USA) program.

### TUNEL assay

To detect apoptosis, the terminal deoxynucleotidyl transferase (TdT) nick end labelling test by the In Situ* Cell Death* detection kit, TMR red (Roche, Mannheim, Germany) was employed as instructed by the manufacturer and described earlier [40].

### Electron microscopy

Tissue processing was performed using a modified protocol [49]. A small piece from the left ventricular (LV) free wall was dissected from the frozen tissue, it was fixated in 2% paraformaldehyde (Sigma Aldrich, St. Louis, MO, USA) and 3% glutaraldehyde (ApplyChem GmbH, Darmstadt, Germany) containing ice-cold phosphate buffered saline (PBS, pH = 7.4) overnight at 4 °C. The tissues were then washed in 0.1 M PBS, osmicated, dehydrated, and embedded into DURCUPAN™ ACM (Sigma Aldrich, St. Louis, MO, USA) resin. 50 nm thin sections were cut from each sample block, contrasted for 1 min with UranyLess (Electron Microscopy Sciences, Hatfield, PA, USA) and 1 min with lead-citrate (Electron Microscopy Sciences, Hatfield, PA, USA). Pictures were captured using a Zeiss LEO 910 electron microscope with magnifications of 4000 × and 8000 × . Densitometry analysis was performed as described earlier, using the ImageJ (National Institutes of Health, Bethesda, Maryland, USA) program [40].

### Force measurements in isolated cardiomyocytes

The technique for isometric force measurements in permeabilised, single cardiomyocyte preparations was described earlier [50–52]. Repeated activation–relaxation cycles were evoked in cardiomyocytes at 15 °C, at a sarcomere length of 2.2 μm. Force values were normalised for the maximal Ca^2+^-activated active force, and Ca^2+^–force relations were fitted to a modified Hill equation to assess the Ca^2+^-sensitivity of isometric force generation, i.e. pCa_50_. The Ca^2+^-dependent active force (F_max_), Ca^2+^-independent passive force (F_passive_) and the rate constant of force redevelopment (*k*_tr,max_) were then evaluated. F_max_ and F_passive_ were normalised for the cross-sectional area of the single cardiomyocytes, which was determined by optically directed light.

### Western immunoblot for the members of the cellular energy sensor system

Myocardial tissue samples from the LV free wall were solubilised and the protein concentration was measured as previously described [40]. Samples were run on 8% SDS–polyacrylamide gel for approximately 1 h at 150 V, using 30 mA/gel. Proteins were transferred onto a nitrocellulose membrane for 90 min at 100 V, using 250 mA. After a 1-h blocking with 1% bovine serum albumin (BSA) in Tris-buffered saline containing 0.1% Tween 20 (TBST), the membranes were probed with anti-peroxisome proliferator-activated receptor-gamma coactivator 1 alpha (PGC1α), phospho- and total anti-acetyl coenzyme A (pACC and ACC) and anti-Forkhead box protein O1 (FoxO1) antibodies in 1% BSA in TBST solution overnight at 4 °C. All primary antibodies were produced by Cell Signaling Technology (Boston, MA, USA). After washing the membranes, incubation was performed with peroxidase-conjugated anti-rabbit IgG-specific antibody (Sigma Aldrich, St. Louis, MO, USA) used in a dilution of 1:40,000. The bands were detected using Westernbright ECL kit (Advansta, San Jose, CA, USA) on a gel documentation system (MF-ChemiBIS, DNR Bio-Imaging Systems). Bands were analysed with the ImageJ (National Institutes of Health, Bethesda, Maryland, USA) program using β-actin labelling (Sigma Aldrich, St. Louis, MO, USA) on the same membrane.

### Western immunoblot for caspase-3

The expression level of caspase-3 in the heart tissue was evaluated using Western immunoblot analysis. The protocol was carried out based on the method reported by Lódi et al. [40]. A total of 35 μg of protein in each sample was loaded and separated on 4–20% Mini-PROTEAN^®^ TGX^™^ Precast Protein Gels (Bio-Rad Laboratories, Hercules, CA, USA), then samples were transferred to a polyvinylidene difluoride (PVDF) membrane (Bio-Rad Laboratories, Hercules, CA, USA). After blocking, the membranes were probed with primary antibodies (caspase-3 1:500, Cell Signaling Technology, Boston, MA, USA). After rinsing, the membranes were incubated with horseradish peroxidase (HRP)-conjugated secondary antibody (1:2000, Cell Signaling Technology, Boston, MA, USA). Finally, to visualize the bands, an enhanced chemiluminescent HRP-substrate was employed. The chemiluminescent bands were normalised to the total protein in each lane with Image Lab™ 5.2.1. Software (Bio-Rad Laboratories, Hercules, CA, USA) [53]. The relative intensity was then compared to an internal control.

### Serum ACE and ACE_2_ activity measurements

Native blood samples were centrifuged at 1500 g for 15 min, and sera were stored at –70 °C. ACE and ACE_2_ activity measurements were carried out based on the previously described studies of our laboratory [54, 55]. Abz-FRK(Dnp)P-OH (Sigma-Aldrich, St. Louis, MO, USA) and Mca-APK(Dnp) (EZ Biolab, Carmel, IN, USA) quenched fluorescent substrates were used to determine the activity of the ACE and ACE_2_.

In the case of ACE activity measurements, the measurement mixture contained 100 mM, pH 7.0 TRIS HCl (Sigma Aldrich), 50 mM NaCl, 10 µM ZnCl_2_, 10 µM Abz-FRK(Dnp)P-OH. For ACE_2_ activity measurements, a protease inhibitor mixture was used, which was composed of 10 µM Bestatin-hydrochloride, 10 µM Z-prolyl-prolinal, (Enzo Life Science, Exeter, UK), 5 µM Amastatin-hydrochloride, 10 µM captopril in a buffer of 500 mM NaCl, 100 µM ZnCl_2_**,** 75 mM TRIS HCl, pH 6.5. The specificity of the activity assays was tested by the specific ACE inhibitor captopril and the specific ACE_2_ inhibitor MLN-4760 (Sigma-Aldrich, St. Louis, MO, USA).

Activity reaction mixtures were set up in a 96 well style, black plates (Greiner-Bio One, Frickenhauser, Germany) at 37 °C. Measurements were performed with a fluorescent plate reader (NOVOstar, BMG Labtech, Ortenberg, Germany) at **λ**_ex_ 340 nm and **λ**_em_ 405 nm for both enzymes. The results were accepted when the goodness of fit (r^2^) was at least 0.90. Activity was calculated using the following equation:

$${\text{ACE}}\;{\text{or}}\;{\text{ACE}}_{{\text{2}}} \;{\text{activity}}\;{\text{ = }}\;{\text{(S/k)}}\;{\text{*}}\;{\text{D,}} $$

where S is the rate of the observed increase in fluorescent intensity (1/min), k is the change in fluorescence intensity upon the complete cleavage of 1 nmole of the fluorescent substrate, and D is the dilution of the sample. ACE and ACE_2_ activities were given in units (U), where 1 U is equivalent to the cleavage of 1 µmole of the fluorescent substrate in 1 min.

### Data analysis and statistics

During the mechanical measurements, Ca^2+^-induced contractions of the isolated cardiomyocytes were recorded with a custom-built LabVIEW Data Acquisition platform. The contractile parameters of the cellular preparations were analysed with the LabVIEW analysing software package (Myo; National Instruments, Austin, TX, USA) and Origin 6.0 (Originlab Corporation, Northampton, MA, USA). The signal intensities of protein bands were analysed using the ImageJ (National Institutes of Health, Bethesda, Maryland, USA) and Magic Plot (Magicplot Systems, Saint Petersburg, Russia) software packages. Variables were measured multiple times, averaged within each animal and used as a single-value characteristic of that animal (“mean of the mean”; except for body weight, body mass index, and strain imaging). The sample sizes of the study groups are indicated in the Figures. The number of measurements is discussed in the Figure and Table legends. Between-groups comparisons for the survival outcome were based on an all-groups log-rank test followed by all possible pairwise variants. For between-groups tests of all other outcomes, analysis of variance or the Kruskal–Wallis test was applied for overall, and Student’s two-sample t test or Wilcoxon’s rank-sum test for pairwise comparisons, as appropriate for normality assumptions on distribution shapes being satisfied or not. Follow-up vs. baseline comparisons within each group were based on paired t tests (normality assumptions satisfied) or Wilcoxon’s matched-pairs signed-ranks tests (otherwise). Values are given as mean ± standard error of the mean (SEM). The criterion for statistical significance was p < 0.05. The statistical package Stata (StataCorp. 2017. Stata Statistical Software: Release 15. College Station, TX: StataCorp LLC) was used for data handling and analysis.

## Results

**Fig. 2:**
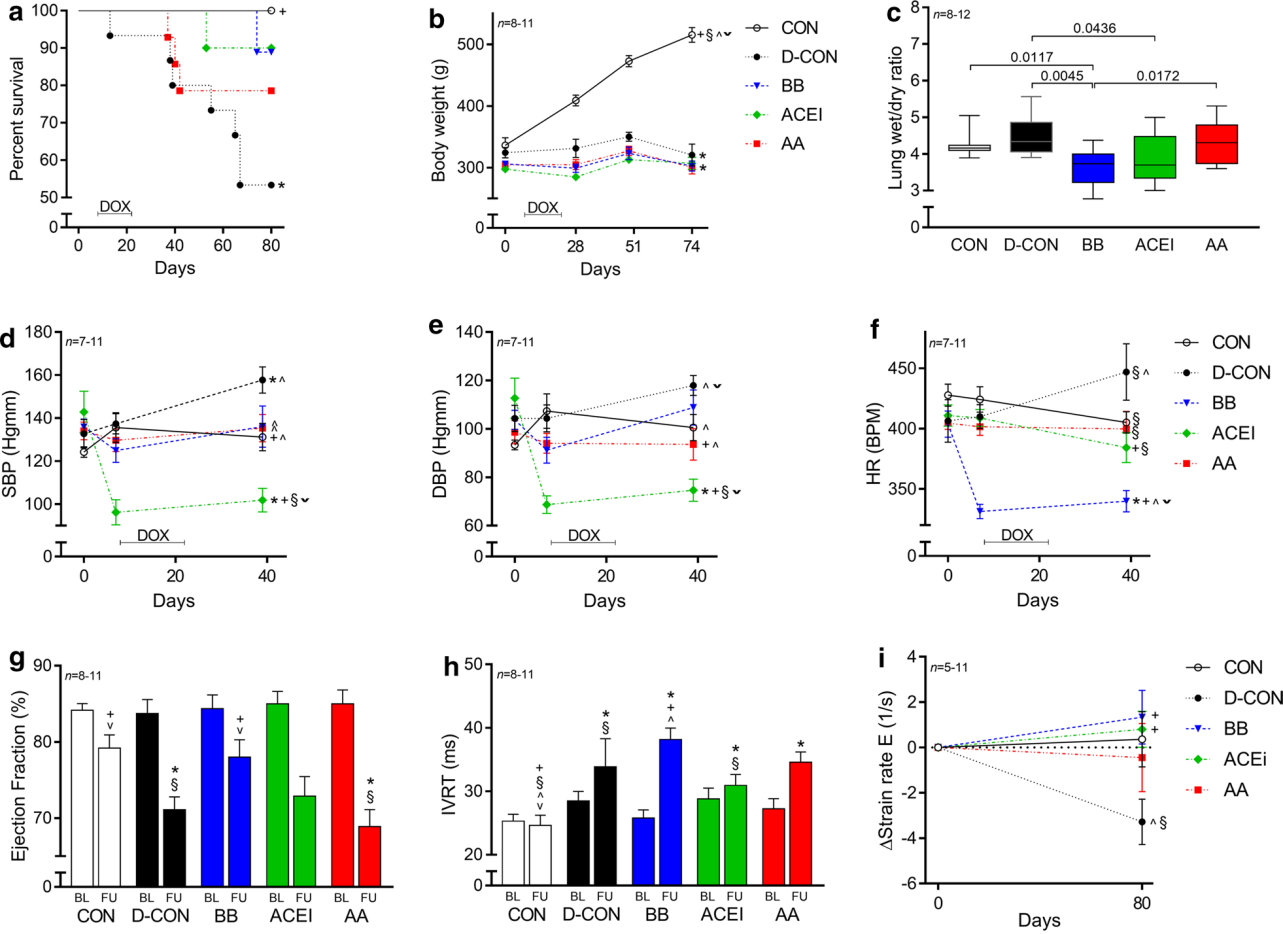
Survival rate, clinical, echocardiographic, and post mortem data of the animals. The survival rate in the D-CON group was significantly worse compared to CON, while both the BB and ACEI treatments resulted in an apparent survival benefit compared to D-CON, which was statistically non-significant with this number of study animals. **a** The growth rate of the animals was significantly higher in the CON group compared to the DOX-receiving groups, irrespective of the applied cardiovascular pre-treatments. **b** Significantly decreased wet/dry ratios were found in the lung samples of the BB and ACEI receiving animals compared to those in the D-CON group. **c** DOX treatment significantly increased the systolic BP of the animals compared to CON. Systolic and diastolic BPs were significantly lower in the ACEI group compared to all other groups. **d, e** HR was significantly lower in the BB group compared to all other groups. **f** A significantly decreased ejection fraction could be observed in the D-CON and AA animals compared to CON, while this parameter remained preserved in the BB group. **g** The IVRT was increased in all DOX-treated animals compared to CON. **h** The change in diastolic strain rate was more pronounced in the D-CON group compared to the BB and ACEI groups. **i** The lines at bottom represent the duration of doxorubicin exposure (DOX). *n* = number of animals per group (5 measurements per animal in case of blood pressure and heart rate, 3 measurements per animal in case of echocardiographic parameters, except for single body weight and strain rate measurements); Statistics: Wilcoxon’s rank-sum tests except for survival (log-rank test); *p < 0.05 vs. CON; ^+^p < 0.05 vs. D-CON; ^§^p < 0.05 vs. BB; ^p < 0.05 vs. ACEI; ˇp < 0.05 vs. AA. BL = baseline, DBP = diastolic blood pressure, FU = follow-up, HR = heart rate, IVRT = isovolumetric relaxation time, SBP = systolic blood pressure

The clinical parameters of the study animals are presented in Table 1. The survival rates of the D-CON animals were significantly lower compared to CON (p = 0.0205), while both the BB and ACEI treatments resulted in an apparent survival benefit compared to D-CON, which was statistically non-significant with this number of study animals [p = 0.0683 (BB vs. D-CON), p = 0.0691 (ACEI vs. D-CON)] (Fig. 2a). The growth rate of the animals was significantly higher in the CON group compared to the DOX-receiving groups, irrespective of the applied cardiovascular pre-treatments (p < 0.0001) (Fig. 2b). Significantly decreased wet/dry ratios were found in the lung samples of the BB and ACEI receiving animals compared to those in the D-CON group [3.67 ± 0.17, 3.89 ± 0.22 vs. 4.51 ± 0.16 in BB, ACEI and D-CON, respectively, p = 0.0045 (BB vs. D-CON), p = 0.0436 (ACEI vs. D-CON)] (Fig. 2c). The BP and HR were monitored until day 39. Afterwards, the tail of the DOX-treated animals became stiff and sclerotic due to the direct toxicity of intravenous DOX, hindering reliable tail-cuff measurements. DOX treatment significantly increased systolic BP of the animals compared to CON (157.69 ± 6.07/117.94 ± 4.08 vs. 131.16 ± 6.39/100.53 ± 5.45 mmHg in D-CON and CON, respectively, p = 0.0129/0.0566) (Fig. 2d, e). Systolic and diastolic BP were significantly lower in the ACEI group compared to all other groups [101.9 ± 5.49/74.65 ± 4.58 vs. 131.16 ± 6.39/100.53 ± 5.45, 157.69 ± 6.07/117.94 ± 4.08, 136 ± 9.65/108.85 ± 7.32, 135.38 ± 6.39/93.62 ± 6.53 mmHg in ACEI and CON, D-CON, BB, AA, respectively, p = 0.0037/p = 0.0027 (ACEI vs. CON), p = 0.0012/p = 0.0012 (ACEI vs. D-CON), p = 0.0083/p = 0.0014 (ACEI vs. BB), p = 0.0015/p = 0.0421 (ACEI vs. AA)] (Fig. 2d, e). HR was significantly lower in the BB group compared to all other groups [339.85 ± 8.83 vs. 405.09 ± 8.87, 446.86 ± 23.42, 384.3 ± 12.36, 399.76 ± 14.64 BPM in BB and CON, D-CON, ACEI, AA, respectively, p = 0.001 (BB vs. CON), p = 0.0026 (BB vs. D-CON), p = 0.011 (BB vs. ACEI), p = 0.0055 (BB vs. AA)] (Fig. 2f).

**Table 1:** Clinical parameters of the animals

	CON (n = 9)			D-CON (n = 7–8)			BB (n = 8)			ACEI (n = 9)			AA (n = 11)		
Days	0	49	74	0	49	74	0	49	74	0	49	74	0	49	74
BW (g)	336.11 ± 12.29§^ˇ	472.89 ± 9.05#^+^§^ˇ	515.44 ± 11.91#†^+^§^ˇ	324.25 ± 8.48^ˇ	350.25 ± 7.38#*§^	320.5 ± 17.87*	306.13 ± 4.19*	323.13 ± 10.21* ^+^	302.75 ± 8.25†*	297.67 ± 4.16* ^+^	313 ± 6.76#*^+^	306.89 ± 9.98*	304.55 ± 4.56*^+^	327.36 ± 7.68#*	300.36 ± 10.43†*
tBMI	154.85 ± 4.42^+^§^	220.85 ± 8.97#^+^^ ˇ	244.1 ± 9.63#†^+^§^ˇ	176.28 ± 6.23*	192.15 ± 6.97#*	179.08 ± 11.6*	194.18 ± 12.15*ˇ	204.67 ± 13.8	192.05 ± 12.82†*ˇ	173.94 ± 7.2*	182.64 ± 7.24#*	179.07 ± 8.43*	164.85 ± 4.91§	177.18 ± 6.26#*	159.67 ± 6.21†*§

	CON (n = 9)			D-CON (n = 7)			BB (n = 8)			ACEI (n = 9)			AA (n = 11)		
Days	0	7	39	0	7	39	0	7	39	0	7	39	0	7	39
SBP	124.22 ± 2.44§	135.64 ± 6.92^	131.16 ± 6.39^+^^	132.74 ± 6.63	137.34 ± 4.67^	157.69 ± 6.07#†*^ˇ	135.88 ± 3.69*	124.95 ± 5.55#^	136 ± 9.65^	142.8 ± 9.7	96.2 ± 5.86#^+^*§ˇ	101.9 ± 5.49#*^+^§ˇ	134.07 ± 4.11	129.67 ± 5.24^	135.38 ± 6.39^+^^
DBP	93.33 ± 2.05§	107.31 ± 7.12§^	100.53 ± 5.45^	104.31 ± 5.44	104.34 ± 5.54^	117.94 ± 4.08#†^ˇ	103.68 ± 3.97*	91.23 ± 5.34#*^	108.85 ± 7.32^	112.7 ± 8.26	68.73 ± 3.67#*^+^§ˇ	74.65 ± 4.58#*^+^§ˇ	98.58 ± 4.79	94.05 ± 4.08^	93.62 ± 6.53^+^^
HR	427.62 ± 9.23ˇ	424.13 ± 10.52§	405.09 ± 8.87†§	406.31 ± 17.7	409.91 ± 9.9§	446.86 ± 23.42§^	403.7 ± 10.82	331.4 ± 5.97#*^+^^ˇ	339.85 ± 8.83#*^+^^ˇ	410.78 ± 8.88	408.85 ± 6.97§	384.3 ± 12.36^+^§	404.45 ± 5.19*	401.51 ± 7.19§	399.76 ± 14.64§

*n* = number of animals per group (5 measurements per animal, except for single body weight and tBMI measurements)

*BW* body weight, *DBP* diastolic blood pressure, *HR* heart rate, *SBP* systolic blood pressure, *tBMI* body mass indexed for tibia length

*p < 0.05 vs. CON; ^+^p < 0.05 vs. D-CON; ^§^p < 0.05 vs. BB; ^p < 0.05 vs. ACEI; ˇp < 0.05 vs. AA; ^#^p < 0.05 vs. day 0; ^†^p < 0.05 vs. day 7 (for SBP, DBP and HR) or day 49 (for BW or tBMI)

Echocardiographic data of the study animals are presented in detail in Table 2. On the follow-up echocardiography, a significantly decreased ejection fraction (EF) could be observed in the D-CON and AA animals compared to CON [71.08 ± 1.69, 68.88 ± 2.23 vs. 79.19 ± 1.73% in D-CON, AA and CON, respectively, p = 0.0046 (D-CON vs. CON), p = 0.0024 (AA vs. CON)], while this parameter remained preserved in the BB group [78 ± 2.28%, p = 0.6304 (BB vs. CON), p = 0.0357 (BB vs. D-CON), p = 0.0117 (BB vs. AA)] (Fig. 2g). Although the EF in the ACEI group demonstrated a significant reduction at follow-up compared to baseline (p = 0.0022), the follow-up EF value was statistically not significantly different from CON with this number of study animals (p = 0.0586) (Fig. 2g). The isovolumetric relaxation time (IVRT) was increased in all DOX-treated animals compared to CON [33.88 ± 4.41, 38.17 ± 1.8, 30.93 ± 1.73, 34.6 ± 1.58 vs. 24.63 ± 1.59 ms in D-CON, BB, ACEI, AA and CON, respectively, p = 0.0343 (D-CON vs. CON), p < 0.0001 (BB vs. CON), p = 0.0165 (ACEI vs. CON), p = 0.0003 (AA vs. CON)] (Fig. 2h). No significant differences could be observed in the change of the systolic strain rate (0.55 ± 0.88, − 1.05 ± 1.1, 0.3 ± 0.68, − 0.65 ± 1.43, − 0.22 ± 0.88 1/s in CON, D-CON, BB, ACEI, AA, respectively; data not shown), while the change in diastolic strain rate was greatest in the D-CON group [− 3.28 ± 1 vs. 0.36 ± 1.22, 1.34 ± 1.18, 0.81 ± 0.79, − 0.44 ± 1.5 1/s in D-CON and CON, BB, ACEI, AA, respectively p = 0.0679 (D-CON vs. CON), p = 0.0152 (D-CON vs. BB), p = 0.025 (D-CON vs. ACEI), p = 0.1914 (D-CON vs. AA)] (Fig. 2i).

**Table 2:** Echocardiographic parameters of the animals

	CON (*n* = 9)		D-CON (*n* = 8)		BB (*n* = 6–8)		ACEI (*n* = 8–9)		AA (*n* = 11)	
	BL	FU	BL	FU	BL	FU	BL	FU	BL	FU
EF (%)	84.15 ± 0.87	79.19 ± 1.73#^+^ˇ	83.71 ± 1.85	71.09 ± 1.69#*§	84.38 ± 1.78	78 ± 2.28^+^ˇ	85 ± 1.62	72.89 ± 2.56#	85 ± 1.83	68.88 ± 2.23#*§
IVSd (mm)	1.09 ± 0.1	1.21 ± 0.10	1.03 ± 0.04	1.26 ± 0.09	1.13 ± 0.03	1.07 ± 0.04	1.09 ± 0.03	1.07 ± 0.03	1.05 ± 0.05	1.05 ± 0.04
IVSs (mm)	1.66 ± 0.22^	1.56 ± 0.1§^ˇ	1.38 ± 0.14^	1.46 ± 0.04§^ˇ	1.16 ± 0.06	1.15 ± 0.04*^+^	1.03 ± 0.03*^+^ˇ	1.09 ± 0.03*^+^	1.16 ± 0.03^	1.08 ± 0.04*^+^
PWd (mm)	0.84 ± 0.03	1 ± 0.04#§^ ˇ	0.82 ± 0.04	1.02 ± 0.03#§^ˇ	0.88 ± 0.03	0.9 ± 0.02*^+^	0.81 ± 0.03	0.81 ± 0.04*^+^	0.85 ± 0.03	0.89 ± 0.02*^+^
PWs (mm)	1.34 ± 0.12^ˇ	1.39 ± 0.1§^ˇ	1.22 ± 0.16	1.37 ± 0.11§^ˇ	1.06 ± 0.02ˇ	0.99 ± 0.04*^+^	0.99 ± 0.05*	1 ± 0.02*^+^	0.95 ± 0.03*§	0.95 ± 0.02*^+^
LVEDD (mm)	6.36 ± 0.25§^ˇ	6.76 ± 0.21§^ˇ	5.59 ± 0.35^	6.13 ± 0.33#^	4.87 ± 0.24*	5.72 ± 0.21*	4.5 ± 0.2*^+^	5.09 ± 0.23#*^+^	4.81 ± 0.25*	5.32 ± 0.26*
LVESD (mm)	3.31 ± 0.15§^ˇ	3.85 ± 0.19#§	2.92 ± 0.21^	3.95 ± 0.26#^	2.53 ± 0.21*	3.32 ± 0.17*	2.3 ± 0.16*^+^	3.21 ± 0.23#^+^	2.44 ± 0.21*	3.5 ± 0.2#
IVCT (ms)	19.41 ± 1.12§	18.18 ± 1.52	19.42 ± 2.58§	22.67 ± 1.93	14.87 ± 0.77*^+^^	20.54 ± 0.88#	18.07 ± 0.99§	18.67 ± 1ˇ	17.52 ± 0.9	22.82 ± 1.76#^
IVRT (ms)	25.3 ± 1.08	24.63 ± 1.59^+^§^ˇ	28.48 ± 1.5	33.88 ± 4.41*§	25.79 ± 1.28	38.17 ± 1.8#*^+^^	28.81 ± 1.67	30.93 ± 1.73*§	27.24 ± 1.59	34.61 ± 1.58#*
DecT (ms)	40.37 ± 1.3	45.56 ± 2.68§^ˇ	37.96 ± 3.58	42.5 ± 2.77^	34.79 ± 3.42	36.63 ± 2.46*	42.04 ± 2.65	33.7 ± 2.1#*^+^	38.15 ± 2.26	37.79 ± 2.3*
ET (ms)	64.7 ± 2.05^+^§^ˇ	62.3 ± 1.47^+^§	59.29 ± 2.39*	81.46 ± 7.77#*^	57.29 ± 3.22*	73.71 ± 1.86#*^	58.59 ± 1.36*	63.93 ± 2.14#^+^§	56.58 ± 1.66*	68.61 ± 2.84#
E (cm/s)	66.63 ± 2.8	64.26 ± 4.21	60.25 ± 4.81	59.63 ± 1.88	63.67 ± 4.81	60.96 ± 4.09	65.33 ± 2.56	58.89 ± 2.81	60.06 ± 2.98	57.55 ± 3.6
A (cm/s)	47.07 ± 4.1	42.26 ± 3.65ˇ	40.21 ± 4.46	37.29 ± 3.22ˇ	43.78 ± 5.32	34.17 ± 2.16ˇ	48.08 ± 4.53	35.75 ± 3.09#ˇ	43.09 ± 3.04	25.06 ± 2.47#*^+^§^
e’ (cm/s)	3.63 ± 0.38	4.44 ± 0.3	3.75 ± 0.25§	4.33 ± 0.35	4.58 ± 0.27^+^^	4.21 ± 0.5	3.59 ± 0.19§	4.44 ± 0.42	4.03 ± 0.28	3.79 ± 0.31
a’ (cm/s)	5.52 ± 0.62	4.41 ± 0.48	4.71 ± 0.39	4.29 ± 0.31	5.57 ± 0.86	4.19 ± 0.71	4.79 ± 0.32	3.88 ± 0.3	5.52 ± 0.55	3.48 ± 0.33#
s’ (cm/s)	27.56 ± 2.38§^ˇ	30.89 ± 1.71	32.67 ± 2.02	29.5 ± 1.6	37.13 ± 1.44*	30.79 ± 1.67#	37.83 ± 1.51*	30.42 ± 1.24#	35.48 ± 2.34*	28.03 ± 1.15#
E/e’	20.11 ± 2.23§	15.09 ± 1.52	16.83 ± 2.06	14.31 ± 1.06	14.25 ± 1.32*^	15.07 ± 0.94	18.53 ± 1.09§	14.32 ± 1.58	15.7 ± 1.48	15.88 ± 1.23
Tei-index	0.7 ± 0.03	0.7 ± 0.05ˇ	0.82 ± 0.09	0.7 ± 0.04ˇ	0.72 ± 0.07	0.8 ± 0.02	0.8 ± 0.04	0.78 ± 0.03	0.79 ± 0.04	0.83 ± 0.04*^+^

A= mitral A wave; a’ = late diastolic velocity of the mitral anulus; BL = baseline; DecT = deceleration time; E = mitral E wave; e’ = early diastolic velocity of the mitral anulus; EF = ejection fraction; ET = ejection time; FU = follow-up; IVCT = isovolumetric contraction time; IVRT = isovolumetric relaxation time; IVSd = diastolic thickness of the interventricular septum; IVSs = systolic thickness of the interventricular septum; LVEDd = end-diastolic diameter of the left ventricle; LVESd = end-systolic diameter of the left ventricle; PWd = diastolic thickness of the posterior wall; PWs = systolic thickness of the posterior wall; s’ = systolic velocity of the mitral anulus; Tei-index = myocardial performance index [(IVCT + IVRT)/ET]. *n* = number of animals per group (3 measurements per animal)

*p < 0.05 vs. CON; ^+^p < 0.05 vs. D-CON; ^§^p < 0.05 vs. BB; ^^^p < 0.05 vs. ACEI; ˇp < 0.05 vs. AA; ^#^p < 0.05 vs. BL

**Fig. 3:**
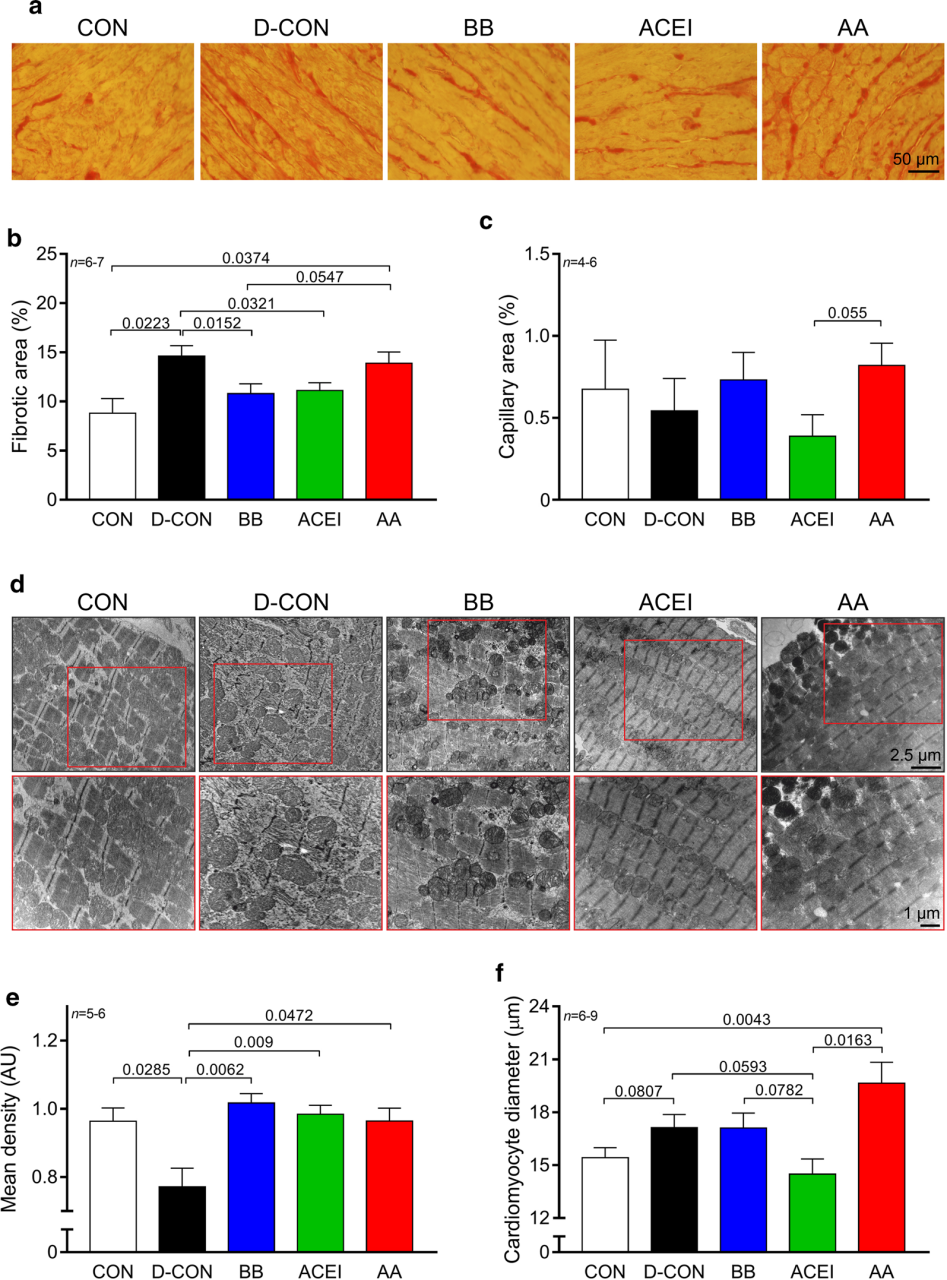
Myocardial fibrosis, capillary density, and electron microscopic imaging. Representative images of myocardial sections stained with picrosirius red and Mayer’s hemalum in all groups. The colour red identifies fibrosis. **a** The fibrotic area in the heart samples was significantly larger in the D-CON and AA groups compared to CON, while the BB and ACEI treatments significantly decreased the level of fibrosis compared to that in the D-CON group. **a, b** When analysing the capillary density of the myocardial sections, no statistically significant differences could be identified between the groups. **c** Electron microscopic images displayed robust ultrastructural changes in the myocardium of the D-CON animals (apoptotic cardiomyocytes, vacuolisation, mitochondrial damage, myofibrillolysis), leading to a decreased overall density of the acquired images compared to CON. Images taken in the groups receiving any prophylactic treatment showed significantly less ultrastructural damage compared to D-CON. **d, e** DOX exposure resulted in a tendency for the development of cardiomyocyte hypertrophy, which was the most apparent in the AA group. The ACEI treatment successfully prevented this change and cardiomyocyte diameters remained small in that group. **f** *n* = number of animals per group (9 images per animal for fibrosis, 1–5 images per animal for capillary density, 2–5 pictures per animal taken at the level of the nucleus for cardiomyocyte diameter measurements, 1–3 pictures per animal for densitometry); Statistics: Wilcoxon’s rank-sum test; numbers are p values. AU = optical density in arbitrary units

The fibrotic area in the heart samples was significantly larger in the D-CON and AA groups compared to CON [14.62 ± 1.06, 13.88 ± 1.16 vs. 8.8 ± 1.51% in D-CON, AA and CON, respectively, p = 0.0223 (D-CON vs. CON), p = 0.0374 (AA vs. CON)], while the BB and ACEI treatments significantly decreased the level of fibrosis compared to that in the D-CON group [10.8 ± 0.99 and 11.12 ± 0.79 in BB and ACEI, respectively, p = 0.0152 (BB vs. D-CON), p = 0.0321 (ACEI vs. D-CON)] (Fig. 3a, b). When analysing the capillary density of the myocardial sections, no statistically significant differences could be identified between the study groups (Fig. 3c). Electron microscopic images displayed robust ultrastructural changes in the myocardium of the D-CON animals: apoptotic cardiomyocytes, vacuolisation, mitochondrial damage, and myofibrillolysis. All these abnormalities led to a decreased overall density of the acquired images compared to CON (0.77 ± 0.05 vs. 0.96 ± 0.04 AU in D-CON and CON, respectively, p = 0.0285) (Fig. 3d, e). At the same time, electron microscopic images taken in the groups receiving any prophylactic treatment showed significantly less ultrastructural damage compared to D-CON, which was also reflected in the densitometry values of these groups [1.02 ± 0.03, 0.98 ± 0.03 and 0.96 ± 0.04 AU in BB, ACEI and AA, respectively, p = 0.0062 (BB vs. D-CON), p = 0.009 (ACEI vs. D-CON), p = 0.0472 (AA vs. D-CON)] (Fig. 3d, e). Nonetheless, some degree of apoptotic activity was also apparent in the images of the groups receiving prophylactic treatments, however, the lower extent of these abnormalities did not substantially compromise either the overall appearance of the ultrastructure, or the densitometry values of the acquired images. Upon DOX exposure, there was a tendency for the development of cardiomyocyte hypertrophy, which was most apparent in the AA group [17.13 ± 0.75, 17.1 ± 0.85, 19.65 ± 1.19 vs. 15.42 ± 0.57 µm in D-CON, BB, AA and CON, respectively, p = 0.0807 (D-CON vs. CON), p = 0.1161 (BB vs. CON), p = 0.0043 (AA vs. CON)] (Fig. 3f). The ACEI treatment successfully prevented this change and cardiomyocyte diameters remained small there [14.49 ± 0.87 µm in ACEI, p = 0.3914 (ACEI vs. CON), p = 0.0593 (ACEI vs. D-CON), p = 0.0782 (ACEI vs. BB), p = 0.0163 (ACEI vs. AA)] (Fig. 3f).

**Fig. 4:**
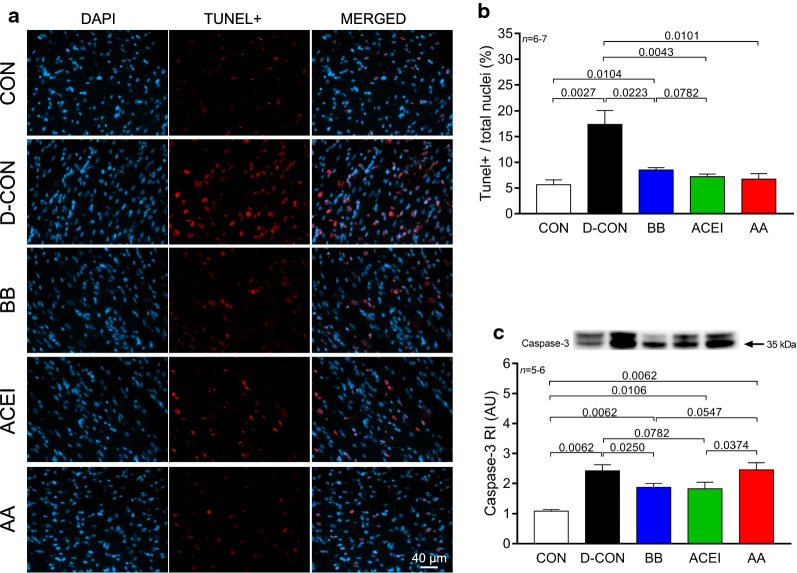
TUNEL assay and caspase-3 levels detecting apoptotic activity. Representative images of cardiomyocyte apoptosis detected by TUNEL. The colour blue denotes all nuclei, the colour red denotes DNA damage, while the colour purple on the merged images denotes nuclei of TUNEL positive cardiomyocytes. **a** Significantly more TUNEL positive nuclei were present in the D-CON animals than in any other group. **a, b** Caspase-3 levels were increased in all DOX-treated groups compared to CON, however, in the BB and ACEI groups, the level of caspase-3 was significantly lower than in D-CON or AA groups. **c** *n* = number of animals per group (12 images per animal for TUNEL, 2–4 measurements per animal for caspase-3); Statistics: Wilcoxon’s rank-sum test; numbers are p values. AU = optical density in arbitrary units

Significantly more TUNEL positive nuclei were present in the D-CON animals than in any other group [17.36 ± 2.72 vs. 5.65 ± 0.92, 8.49 ± 0.44, 7.19 ± 0.53, 6.71 ± 1.09% in D-CON and CON, BB, ACEI, AA, respectively, p = 0.0027 (D-CON vs. CON), p = 0.0223 (D-CON vs. BB), p = 0.0043 (D-CON vs. ACEI), p = 0.0101 (D-CON vs. AA)] (Fig. 4a, b). Caspase-3 levels were increased in all DOX-treated groups compared to CON [2.42 ± 0.21, 1.87 ± 0.13, 1.83 ± 0.21, 2.46 ± 0.24 vs. 1.09 ± 0.05 AU in D-CON, BB, ACEI, AA and CON, respectively, p = 0.0062 (D-CON, BB or AA vs. CON), p = 0.0106 (ACEI vs. CON)], however, in the BB and ACEI groups, the level of caspase-3 was significantly lower than in D-CON or AA [p = 0.025 (BB vs. D-CON), p = 0.0547 (BB vs. AA), p = 0.0782 (ACEI vs. D-CON), p = 0.0374 (ACEI vs. AA)] (Fig. 4c).

**Fig. 5:**
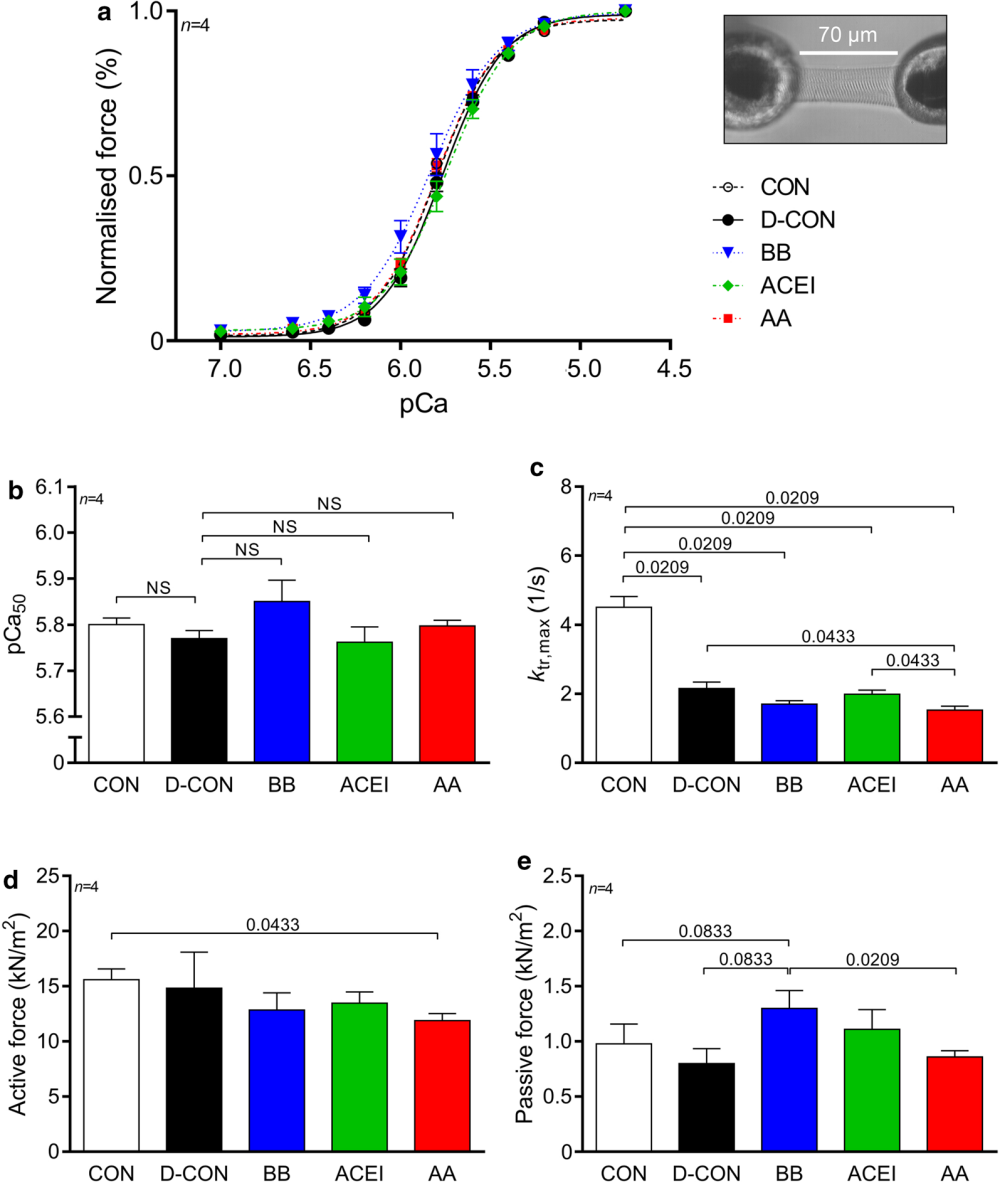
Force measurements in isolated cardiomyocytes. Cardiomyocyte measurements revealed no significant changes in the Ca^2+^-sensitivity (pCa_50_) among groups. **a, b** A smaller *k*_tr,max_ parameter was seen in all DOX-treated animals. **c** Cardiomyocytes isolated from the AA group had a slightly but significantly lower active force value than in CON (**d**), while cardiomyocytes in the BB group had a slightly elevated passive force value. **e** Illustration of an isolated cardiomyocyte. *n* = number of animals per group (2–3 cardiomyocytes per animal); Statistics: Wilcoxon’s rank-sum test; numbers are p values. pCa_50_ = Ca^2+^-sensitivity of isometric force production, *k*_tr,max_ = rate constant of force redevelopment

Isolated, skinned cardiomyocyte measurements revealed no significant changes in the Ca^2+^-sensitivity among groups (Fig. 5a, b, Table 3), while a smaller *k*_tr,max_ parameter could be observed in all DOX-treated animals, irrespective of any prophylactic treatment [2.16 ± 0.19, 1.7 ± 0.1, 1.99 ± 0.11, 1.53 ± 0.11 vs. 4.51 ± 0.31 1/s in D-CON, BB, ACEI, AA and CON, respectively, p = 0.0209 (D-CON, BB, ACEI or AA vs. CON)] (Fig. 5c, Table 3). Cardiomyocytes isolated from the AA group had a slightly but significantly lower active force value than in CON (11.88 ± 0.64 vs. 15.59 ± 1 kN/m^2^ in AA and CON, respectively, p = 0.0433) (Fig. 5d, Table 3), while cardiomyocytes in the BB group had a slightly elevated passive force value [1.3 ± 0.16 vs. 0.97 ± 0.18, 0.8 ± 0.14, 0.86 ± 0.06 kN/m^2^ in BB and CON, D-CON, AA, respectively, p = 0.0833 (BB vs. CON or D-CON), p = 0.0209 (BB vs. AA)] (Fig. 5e, Table 3).

**Table 3:** Contractile parameters of isolated cardiomyocytes

	CON (*n* = 4)	D-CON (*n* = 4)	BB (*n* = 4)	ACEI (*n* = 4)	AA (*n* = 4)
*F*_*max*_ (kN/m^2^)	15.59 ± 0.86ˇ	14.82 ± 2.84	12.85 ± 1.35	13.46 ± 0.88	11.88 ± 0.56*
*F*_*passive*_ (kN/m^2^)	0.98 ± 0.16	0.8 ± 0.12	1.3 ± 0.14ˇ	1.11 ± 0.16	0.86 ± 0.05§
*pCa*_*50*_	5.8 ± 0.01	5.77 ± 0.02	5.85 ± 0.04	5.76 ± 0.03	5.8 ± 0.01
*k*_tr,max_ (1/s)	4.51 ± 0.27^ +^ §^ˇ	2.16 ± 0.16*ˇ	1.7 ± 0.09*	1.99 ± 0.1*ˇ	1.53 ± 0.1*^+^^
*nHill*	2.74 ± 0.22	2.6 ± 0.06	2.31 ± 0.07	2.42 ± 0.17	2.4 ± 0.06

F_max_ = maximum Ca^2+^-activated active force; F_passive_ = Ca^2+^-independent passive force; pCa_50_ = Ca^2+^-sensitivity of isometric force production; *k*_tr,max_ = rate constant of force redevelopment; nHill = steepness of the force–pCa curve characterising the cooperativity between myofilament units. *n* = number of animals per group (2–4 cardiomyocytes per animal)

*p < 0.05 vs. CON; ^+^p < 0.05 vs. D-CON; ^§^p < 0.05 vs. BB; ^p < 0.05 vs. ACEI; ˇp < 0.05 vs. AA

**Fig. 6:**
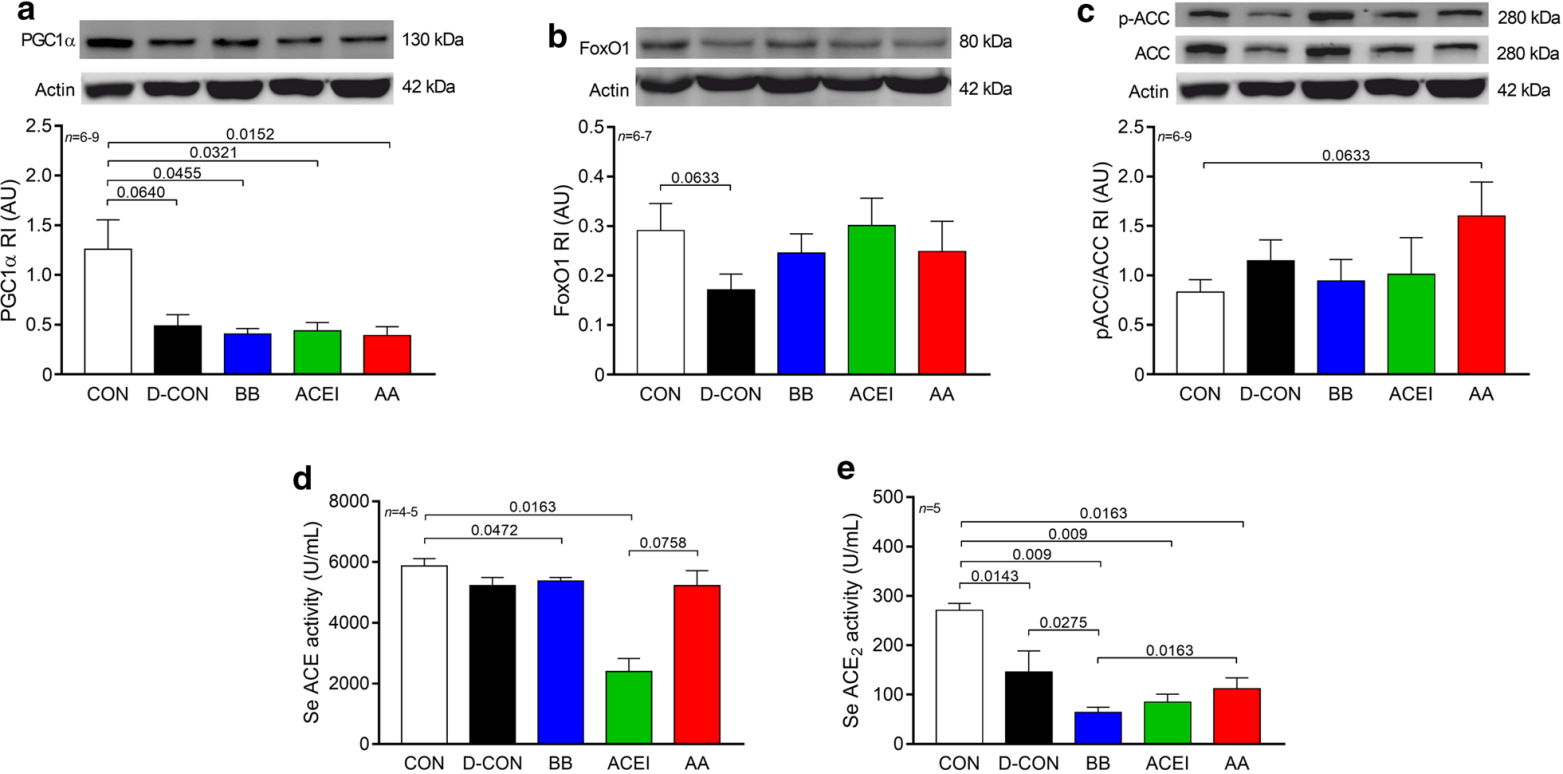
Biochemical measurements of proteins and serum angiotensin-converting enzyme activities. A marked decrease of the PGC1α levels could be detected in DOX-treated animals compared to CON. **a** FoxO1 levels appeared to be lower in the D-CON group compared to CON, however, this change did not reach statistical significance. **b** No significant changes were recognisable in the pACC/ACC levels between the groups. **c** Serum ACE measurements revealed a significantly lower ACE activity in the ACEI group compared to CON, and a numerically less different, but significantly decreased value in the BB group. **d** A robust decrease in the serum ACE_2_ levels of the DOX-exposed animals could be seen compared to healthy controls. **e** *n* = number of animals per group (1–4 bands/animal for Western immunoblots, 2 measurements/animal for ACE and ACE2 activities); Statistics: Wilcoxon’s rank-sum test; numbers are p values. ACC = acetyl coenzyme A carboxylase, AU = optical density in arbitrary units, FoxO1 = Forkhead box protein O1, P–ACC = phosphorylated ACC, PGC1α = peroxisome proliferator-activated receptor-gamma coactivator 1 alpha

In order to reveal the possible changes in the underlying molecular pathophysiology of the mitochondrial damage seen on electron microscopy, we made efforts to assess the members of the cellular energy sensor web, PGC1α and adenosine monophosphate-activated protein kinase activity (by assessing the phosphorylation of ACC, its substrate), as well as FoxO1. Western immunoblot experiments showed a marked decrease of the PGC1α levels in DOX-treated animals compared to CON [0.49 ± 0.11, 0.41 ± 0.05, 0.44 ± 0.08, 0.39 ± 0.09 vs. 1.26 ± 0.3 AU in D-CON, BB, ACEI, AA and CON, respectively, p = 0.064 (D-CON vs. CON), p = 0.0455 (BB vs. CON), p = 0.0321 (ACEI vs. CON), p = 0.0152 (AA vs. CON)] (Fig. 6a). FoxO1 levels appeared to be lower in the D-CON group compared to CON, however, this change did not reach statistical significance (0.17 ± 0.03 vs. 0.29 ± 0.05 AU in D-CON and CON, respectively, p = 0.0633) (Fig. 6b). No significant changes were recognisable in the pACC/ACC levels between the groups (Fig. 6c).

Serum ACE measurements revealed a significantly lower ACE activity in the ACEI group compared to CON (2398 ± 426 vs. 5867 ± 248 U/mL in ACEI and CON, respectively, p = 0.0163), and a numerically less different, but significantly decreased value in the BB group [5379 ± 117 U/mL in BB, p = 0.0472 (BB vs. CON)] (Fig. 6d). In addition, a robust decrease in the serum ACE_2_ levels of the DOX-exposed animals could be seen compared to healthy controls [145 ± 43, 64 ± 10, 85 ± 16, 112 ± 22 vs. 271 ± 14 U/mL in D-CON, BB, ACEI, AA and CON, respectively, p = 0.0143 (D-CON vs. CON), p = 0.009 (BB or ACEI vs. CON), p = 0.0163 (AA vs. CON)] (Fig. 6e).

## Discussion

Myocardial dysfunction and heart failure are well-known and serious complications of AC chemotherapy. As of today, no widespread, routine cardioprotection has been recommended for the primary prevention of AC-associated LV dysfunction in patients at low risk of cardiovascular disease [2, 3, 7, 8]. Results have been controversial in prior human studies, where mainly patient survival, echocardiographic, and blood biomarker data were evaluated, with less emphasis put on the possible mechanisms of cardioprotection [31, 33, 34, 56–58]. This necessitated the development of appropriate pre-clinical models of AC cardiomyopathy in order to examine cardiotoxic side effects and potential preventive strategies both at the in vivo an in vitro levels, with the inclusion of histological, cellular, and molecular explorations. Recently, we have demonstrated preserved LV EF, conserved myocardial ultrastructure, lower apoptosis rate, and improved survival using a triple-combined prophylactic vs. conventionally scheduled cardioprotective therapy in a translational rat model of DOX cardiotoxicity [40]. In the current study, we investigated the individual contribution of the same 3 drug agents (BB, ACEI or AA) to the previously observed beneficial effects. Our main findings are the followings: (1) the LV EF was best preserved by the BB treatment, (2) the myocardial ultrastructure was successfully conserved by all 3 drugs, (3) the DOX-induced higher apoptotic activity could be partially reduced by the BB or ACEI treatments, and (4) all 3 agents contributed to the mortality benefit, with the highest survival rate observed in the BB and ACEI groups.

According to the latest guideline of the European Society of Cardiology, BBs, ACEIs, and AAs are all IA recommendations of pharmacological therapy for HF with reduced EF [59]. The BB bisoprolol, the ACEI perindopril, and the AA eplerenone were previously proven to decrease the risk of hospitalisation and death in clinical studies [60–62]. As DOX cardiotoxicity also contributes to a higher hospitalisation rate, decreased quality of life, and lower survival rate [3, 63–65], the administration of these agents certainly leads to better clinical outcomes, once manifest HF has been detected in the oncological patient. Lately, we have shown that the prophylactic combination of these 3 drugs effectively decreases mortality and prevents myocardial deterioration in a translational rat model [40]. Interestingly, our current work confirms that all 3 agents contribute to the previously observed mortality benefit, with the most favourable results in the BB and ACEI groups. We observed distinct haemodynamic effects both in the case of bisoprolol (decreased HR) and perindopril (decreased BP), which may have compensated for the activation of the adrenergic and renin–angiotensin–aldosterone system, usually seen in HF. On the other hand, no significant haemodynamic consequence of the eplerenone treatment could be seen in the animals at the applied dose (6.25 mg/kg).

Previously, several smaller clinical trials [31–34, 57, 58, 66] and some meta-analyses [67–70] have investigated the prophylactic effects of BBs and ACEIs applied alone or in combination to prevent AC cardiotoxicity, however, their results were not always consistent. Although some study groups demonstrated the protective effects of the applied agent on the LV EF [31, 33, 34, 66], others could not confirm the superiority of the study drug compared to placebo [32, 57, 58]. According to our findings, the best effect on EF could be achieved by the BB treatment, while the AA treatment had no beneficial effect in terms of preventing systolic LV dysfunction. Lately, both human and animal studies have demonstrated a positive effect of the AA spironolactone on LV EF in AC-induced cardiomyopathy [35, 71], nevertheless, recent experimental studies with eplerenone have led to conflicting results [72, 73]. Similarly, the eplerenone applied in our study was insufficient to prevent the development of LV dysfunction. In contrast with prior clinical trials, where the authors found no change in the IVRT in the BB-treated or placebo groups [31, 33], in our model, we consistently identified the increase of this parameter in all DOX-treated groups, independent of any drug treatment – alone or in combination [40]. Still, the virtually unchanged diastolic strain rate seen in the BB or ACEI groups suggests that the DOX-induced impairment of LV relaxation can be mitigated by both of these agents.

The basis of the pathomechanism behind DOX cytotoxicity is the complex forming effect of DOX with topoisomerase II beta (Top2β), causing the inhibition of the enzyme [74–76]. Consequently, this leads to DNA double band break, mitochondrial dysfunction, decreased levels of PGC1α, and decreased mitochondrial biogenesis, eventually resulting in cellular dysfunction and cell death [74, 75]. These events are not only attributed to the primary antineoplastic effects of DOX, but to its cardiotoxic nature as well (representing an on-target side effect). In our study, we found decreased myocardial PGC1α levels, which could not be affected by any of the treatments. Interestingly, this finding may also be implicated as the drugs used in our study are likely to have no significant effects on the antitumor activity of DOX. Future investigations may be able to confirm this hypothesis. Another mechanism of DOX toxicity is the production of reactive oxygen species via the quinone ring of the molecule (off-target side effect), which leads to lipid peroxidation, DNA damage, and protein carbonylation without binding to the Top2β [74]. Furthermore, the pro-inflammatory cytokine-producing effect of DOX is also well-described. Cell cultures and animals treated with DOX have elevated interleukin (IL)-1β, IL-6, IL-8, IL-10 and tumor necrosis factor-α levels [77–80]. Moreover, ILs have been previously shown to be associated with atherosclerosis, an increased risk of myocardial infarction, as well as congestive heart failure [77]. Recently, DOX has also been attributed to a new type of programmed cell death, namely pyroptosis, which is again characterised by pro-inflammation and, among others, increased levels of caspase-1, 3, 4, 5, 11 and IL-1β [81, 82]. In the myocardium, all of the above pathways may result in cardiomyocyte apoptosis, myocardial fibrosis, LV dysfunction and, ultimately, chronic HF, as well as increased mortality. Based on our results, the application of BB, ACEI, or AA significantly decreased the number of apoptotic nuclei and led to a preserved myocardial ultrastructure. In addition, the BB and ACEI treatments substantially decreased the DOX-induced fibrotic remodelling and elevated levels of caspase-3, which enzyme plays a key role in the workings of the apoptotic pathway. Despite the lower FoxO1 levels found in the D-CON group, our analysis did not reveal any significant changes in the myocardial energy stress levels.

We have recently shown that DOX can reduce the rate of the actin-myosin cross-bridge redevelopment [40]. In accordance with our previous findings, here we also detected a markedly decreased *k*_tr,max_ value in all DOX-exposed animals independent of any drug treatment. Furthermore, the increasing tendency of the cardiomyocyte passive stiffness in the BB group could be the result of lower PKA-mediated titin phosphorylation in these cellular preparations. Although mainly non-significant changes could be observed in the cardiomyocyte diameters of the individual groups, our data suggest an antihypertrophic effect of the ACEI treatment. Both a direct drug effect and indirectly, the lower BP of these animals could be responsible for this phenomenon. The circulating serum ACE activity could be effectively inhibited by the ACEI treatment of the animals, which is the basis of renin–angiotensin–aldosterone system inhibition during HF therapy. On the other hand, the robust decrease in serum ACE_2_ activity in the DOX-treated animals may facilitate the bioaccumulation of angiotensin II, which hypothetically could contribute to the deteriorating cardiovascular state of these animals via increased afterload, fibrotic remodelling, and cardiomyocyte hypertrophy. The detailed analysis of the crosstalk between these pathways and the possible impact of the applied treatments on tissue ACE activities were beyond the scope of our current study and will certainly require further investigations.

## Conclusions

In the present work, we investigated the individual effects of prophylactic bisoprolol, perindopril, and eplerenone in an experimental model of DOX cardiomyopathy. According to our results, bisoprolol and perindopril were both effective in mitigating the DOX-induced adverse myocardial changes and increased mortality. The overall performance of eplerenone in our study was only moderate and inferior compared to the other two agents. Taken together, our results suggest that both bisoprolol and perindopril, or preferably the combination of these drugs could potentially attenuate DOX cardiotoxicity when commenced before the chemotherapy. The potential benefits of the translation of these results into clinical practise is still to be further investigated. Nevertheless, the deeper understanding of the mechanisms behind the pharmacological interventions of DOX cardiotoxicity will certainly contribute to a better identification of patients in need of protective measures prior to their oncotherapy.

### Limitations of the study

Despite paying meticulous attention to closely mimicking human pathology in our translational animal model, every experimental setting implies its own limitations concerning the clinical implementation of the findings. Firstly, the pharmacokinetics and pharmacodynamics of rats are different compared to those of humans, which explains the relatively higher doses of oral medications used in our study. As the main reason for the lack of routine prophylactic cardioprotection prior to AC chemotherapy in the clinical setting is the potential development of hypotension, future investigations are more than necessary to confirm the observed beneficial effects by using drug doses also applicable in humans. As opposed to this, the overall cumulative DOX dose applied in our study was lower compared to human protocols; however, the time period between the consecutive cycles were also shorter. Secondly, although female gender is a risk factor in the development of DOX cardiomyopathy in a clinical setting, it is mainly accepted that male rodents are more sensitive to AC exposure compared to females. In our study, solely male rats were used in order to standardise our observations independent of hormonal influences. Lastly, we modelled DOX cardiomyopathy in healthy rats free of comorbidities and oncological disease, making the observation of isolated DOX effects possible. In the future, more sophisticated animal models representing tumor development, undergoing complex radiotherapy and combined chemotherapy would be more suitable to mirror the clinical scenario of oncological patients as closely as possible.

## Data Availability

The datasets generated and analysed during the current study are available in the repository of the University of Debrecen. Access to the datasets is available from the corresponding author on reasonable request.

## Abbreviations

AA: Aldosterone antagonist/eplerenone-receiving group
AC: Anthracycline
ACC: Acetyl coenzyme A carboxylase
ACE: Angiotensin-converting enzyme
ACEI: Angiotensin-converting enzyme inhibitor/perindopril-receiving group
ACE_2_: Angiotensin-converting enzyme 2
AU: Optical density in arbitrary units
BB: β-blocker/bisoprolol-receiving group
BP: Blood pressure
BPM: Beats per minute
BSA: Bovine serum albumin
CON: Negative control group
D-CON: Positive control group
DOX: Doxorubicin
F_max_: Maximum Ca^2^^+^-activated active force
FoxO1: Forkhead box protein O1
F_passive_: Ca^2^^+^-independent passive force
HF: Heart failure
HR: Heart rate
HRP: Horseradish peroxidase
IL: Interleukin
*k*_tr,max_: Rate constant of force redevelopment
LV: Left ventricular
pACC: Phospho-acetyl coenzyme A carboxylase
pCa_50_: Ca^2^^+^-sensitivity of isometric-force production
PBS: Phosphate buffered saline
PGC1α: Peroxisome proliferator-activated receptor-gamma coactivator 1 alpha
PVDF: Polyvinylidene difluoride
SDS: Sodium dodecyl sulphate
SEM: Standard error of the mean
TBST: Tris-buffered saline with Tween 20
Tdt: Terminal deoxynucleotidyl transferase
Top2β: Topoisomerase II beta
